# Institutional Notes and News

**Published:** 1909-10-16

**Authors:** 


### Institutional Notes and News.

### NORFOLK AND NORWICH HOSPITAL.

Upwards of £400 was subscribed at a public meeting held at Norwich last week on behalf of the fund for the extension of the Norfolk and Norwich Hospital. The chief requirements are a new isolation block, a separate building for septic surgical cases, two new operating theatres, an enlargement of the out-patient department, and in conjunction with the Norwich Eye Infirmary, a new block for ophthalmic cases. The sum needed is about £25,000, and a further sum of like amount is required for endowment. Towards this £14,000 had before yesterday's meeting been subscribed. The King has shown his approval of and interest in the scheme by contributing 250 guineas, and he has also signified his intention of laying the foundation stone of the new isolation and special block on the occasion of his visit to the city on October 25.

### NATIONAL HOSPITAL FOR THE PARALYSED AND EPILEPTIC.

On Friday, October 8, the Duchess of Albany, accompanied by Prince and Princess Alexander of Teck, attended an entertainment in aid of the fund for the National Hospital for the Paralysed and Epileptic, Queen Square, Bloomsbury. The entertainment was held at the house of Mr. J. Bland Sutton, and was attended by many friends of the hospital, and a sum of 200 guineas was realised, completing the total of £1,000 required for the special purse, which was presented on the following Saturday at the presentation of purses on behalf of the institution. On that day H.R.H. the Princess of Wales visited the hospital and received fifty purses containing donations for the Jubilee Fund. Mr. Frederic McMillan, in the course of an address, pointed out that the hospital supported 200 beds and a convalescent home, in which over a thousand patients were annually treated, apart altogether from the out-patients. The Jubilee of the hospital will be celebrated early in November next, when his Majesty the King has consented to open the new buildings.

### KENSINGTON AND FULHAM GENERAL HOSPITAL.

In accordance with the Articles of Association, a special meeting of Governors will be held at the Hospital, Richmond Road, Earl's Court, London, S.W., on Saturday, October 16, 1909, at 3 p.m., to consider :—1. Whether the Hospital should place itself unreservedly in the hands of the "King Edward's Hospital Fund for London"; or, 2. Whether the Management should endeavour to make further efforts to carry on its work, as at present; and, if so, 3. What steps should be taken?

### WEST BROMWICH HOSPITAL.

At the annual meeting last week the Chairman, Mr. F. Dudley Docker, proposed a resolution whereby after January 1 next four notes will be issued per one guinea subscribed, no patients receiving immediate treatment under by-laws 15 to receive treatment for a period exceeding 48 hours from admission without obtaining a hospital note, except that in case of an in-patient the treatment may be continued with the consent of the Board of Management. In doing so Mr. Docker observed that some people seemed to regard their subscription more in the light of an investment than a donation to charity. That should not be the case. It was difficult enough to supply even four notes for one guinea, and the resolutions were the outcome of a conference between the Board of Management and the representatives of the various Hospital Saturday and Sunday Committees. The Rev. M. M. Connor seconded, and Mr. Jones, in supporting, said he hoped the effect would be to reduce the number of trivial cases that came up to the out-patient department for treatment. The resolution was unanimously adopted.

### GLASGOW VICTORIA INFIRMARY.

The Glasgow University and the Glasgow Victoria Infirmary have each been promised a gift of £10,000 from the estate of the late Dr. Robert Pollok. His will contained no bequest, but separate writing was found suggesting that the residuary legatees should give £10,000 for the endowment of a special ward in the infirmary and £10,000 for the endowment of a University lectureship for original research in materia medica.

### WORTHING SANATORIUM.

The new ward pavilions of the Worthing Corporation Infectious Diseases Hospital at Swandean were formally opened by the Mayor (Councillor J. G. Denton) last week. Each pavilion contains three wards, with total accommodation (based upon the Local Government Board's requirements as to air space) for twenty-eight beds. A nurses' duty-room is provided, with inspection windows overlooking each ward. There is also a receiving-room, with movable bath, in addition to the usual detached bathrooms and lavatories. One of the new wards will be set apart for diphtheria cases and the other for scarlet fever. An isolation and observation pavilion has been erected, containing a nurses' duty-room and two wards, each accommodating two beds. The whole of the buildings, which may justly be styled "up to date," have been erected from the plans and under the superintendence of the Borough Surveyor (Mr. F. Roberts), the builders of the various sections being Messrs. James Longley and Co., of Crawley; Messrs. Blaker and Son, Worthing; Mr. W. East, and Mr. G. Baker, jun., Worthing. The cost of the new wards and isolation blocks has been about £6,000, and, including the original building and the purchase of all the necessary land required, the total cost of the hospital has been £10,000.

### MANCHESTER INFIRMARY.

At the last monthly meeting the General Secretary reported that a notification had been received that the late Rev. Edward Llewellyn Adams had bequeathed £4,000 to the institution in consideration of a ward being named "The Elizabeth Adams Ward." The amount was originally £4,500, but Mr. Adams, it was stated, "had been compelled to reduce the amount in consequence of the increased duties entailed by the Budget."

### RECOGNITION OF SERVICE.

Mr. Charles Hopkinson, at the last board meeting of the Manchester Infirmary, proposed the adoption of the minutes of the Re-building Committee in which it was recommended that Mr. A. Turner, principal clerk of works, be paid a sum of £400 to be charged to the re-building account in recognition of his exceptional devotion to the interests of the infirmary during the erection of the new hospital, of the efficiency with which he carried out his duties, and the valuable extra services which he had rendered in pricing quantities and adjusting the various accounts. The Committee recognised also the services in regard to furnishing and the arrangements for the King's visit, which involved much overtime. The vote was confirmed.

### BROMYARD WORKHOUSE.

In the medical officer's report for the half-year it was reported that there was not sufficient accommodation at the workhouse for the female sick, and at the last Guardians' meeting, on the motion of the vice-chairman, the matter was referred to the Visiting Committee for their consideration and report. Mr. Mitchell took exception to such a procedure, and remarked that as they were now about to call upon the ratepayers to provide accommodation for the sick inmates, he thought an investigation of the House was necessary, as he was not at all satisfied that private apartments were as they should be. Mr. Mitchell said he did not move a resolution, but he considered it his duty to bring the Board's attention to this matter.

### JOHANNESBURG HOSPITAL.

Under the new constitution of the Johannesburg Hospital, Dr. Mackenzie now takes control of the entire management, under the title of "Superintendent" instead of "Medical Superintendent." The position of secretary is abolished, and his duties merged in those of the superintendent, who is assisted by a clerical staff and an accountant.

### CROYDON ISOLATION HOSPITAL.

The application of the Croydon Rural District Council for sanction to a loan of £4,800 to extend the isolation hospital at Beddington Corner has been the subject of a recent Local Government Board inquiry. The original proposal with regard to the extension of the hospital provided for an expenditure of £7,370, but the Board suggests that the scheme be cut down, and that a conference be held with the medical and sanitary authorities with a view to reducing the scheme. The amended scheme provides for the provision of a new twelve-cubicle observation pavilion, and it is proposed to provide for the first time a cottage for the engineer and another for the gardener.

### SALISBURY INFIRMARY.

The annual thanksgiving service in connection with Salisbury Infirmary was held at the cathedral on Tuesday last week. The collection amounted to £69 3s. 7d., which, although less than last year, was considered good, in view of such unfavourable weather. The observance of this custom dates back to the foundation of the hospital. Last Tuesday's annual service was the 142nd that has been held, and the ancient staff carried in the procession bears the date 1767.

### CARDIFF MENTAL HOSPITAL.

At a recent meeting of the Cardiff Mental Hospital Committee, Messrs. Oatley and Skinner, the architects, wrote that they had made a mistake in estimating for the farm machinery necessary. They had put the figure at £79 6s., but the machinery (chaffcutter, motor, &c.) was more complicated than they had thought, and they, therefore, furnished a new estimate at £120 8s. 5d. Messrs. King and Son wrote accepting the council's offer (as already reported) in settlement of their claim against the corporation regarding their contract for the erection of the mental hospital and the complications which had ensued in regard to the wiring sub-contract. The Chairman said he thought they were in a position to congratulate themselves on being able to come to such an amicable arrangement. Those who were on the old committee felt that the complications which arose might involve them in a big claim over the wiring sub-contract. The original contract was for £235,000. They were able to save something like £5,000 by omitting the isolation hospital, and they were able to complete the contract for £226,000, including any claim that had been made against them. The contractors claimed for £18,000, and they (the contractors) would have been able to get at least £9,000 or £10,000.

### UNIVERSITY COLLEGE.

The following elections to scholarships tenable at University College have been made :—To the Bucknill Scholarship of 135 guineas, Mr. C. I. de Silva, formerly of the Royal College, Colombo, and Medical College, Ceylon; to the Medical Entrance Exhibition of 55 guineas, Mr. R. J. Clausen, formerly of University College School; to the Epsom Free Scholarship, Mr. J. H. Stewart, of Epsom College.

Sir James Crichton-Browne, M.D., F.R.S., will preside at the introductory lecture to the Chadwick training course in hygiene for school teachers. The introductory lecture will be open to the public, and will be delivered at University College by Professor H. R. Kenwood on Wednesday, October 6, at 7.

